# Small molecule autoencoders: architecture engineering to optimize latent space utility and sustainability

**DOI:** 10.1186/s13321-024-00817-0

**Published:** 2024-03-05

**Authors:** Marie Oestreich, Iva Ewert, Matthias Becker

**Affiliations:** Modular High-Performance Computing and Artificial Intelligence, German Center for Neurodegenerative Diseases (DZNE), Bonn, Germany

**Keywords:** Molecular autoencoders, Latent space optimization, Sustainability, Resource optimization

## Abstract

Autoencoders are frequently used to embed molecules for training of downstream deep learning models. However, evaluation of the chemical information quality in the latent spaces is lacking and the model architectures are often arbitrarily chosen. Unoptimized architectures may not only negatively affect latent space quality but also increase energy consumption during training, making the models unsustainable. We conducted systematic experiments to better understand how the autoencoder architecture affects the reconstruction and latent space quality and how it can be optimized towards the encoding task as well as energy consumption. We can show that optimizing the architecture allows us to maintain the quality of a generic architecture but using 97% less data and reducing energy consumption by around 36%. We additionally observed that representing the molecules as SELFIES reduced the reconstruction performance compared to SMILES and that training with enumerated SMILES drastically improved latent space quality. **Scientific Contribution:** This work provides the first comprehensive systematic analysis of how choosing the autoencoder architecture affects the reconstruction performance of small molecules, the chemical information content of the latent space as well as the energy required for training. Demonstrated on the MOSES benchmarking dataset it provides first valuable insights into how autoencoders for the embedding of small molecules can be designed to optimize their utility and simultaneously become more sustainable, both in terms of energy consumption as well as the required amount of training data. All code, data and model checkpoints are made available on Zenodo (Oestreich et al. Small molecule autoencoders: architecture engineering to optimize latent space utility and sustainability. Zenodo, 2024). Furthermore, the top models can be found on GitHub with scripts to encode custom molecules: https://github.com/MarieOestreich/small-molecule-autoencoders.

**Graphical Abstract:**

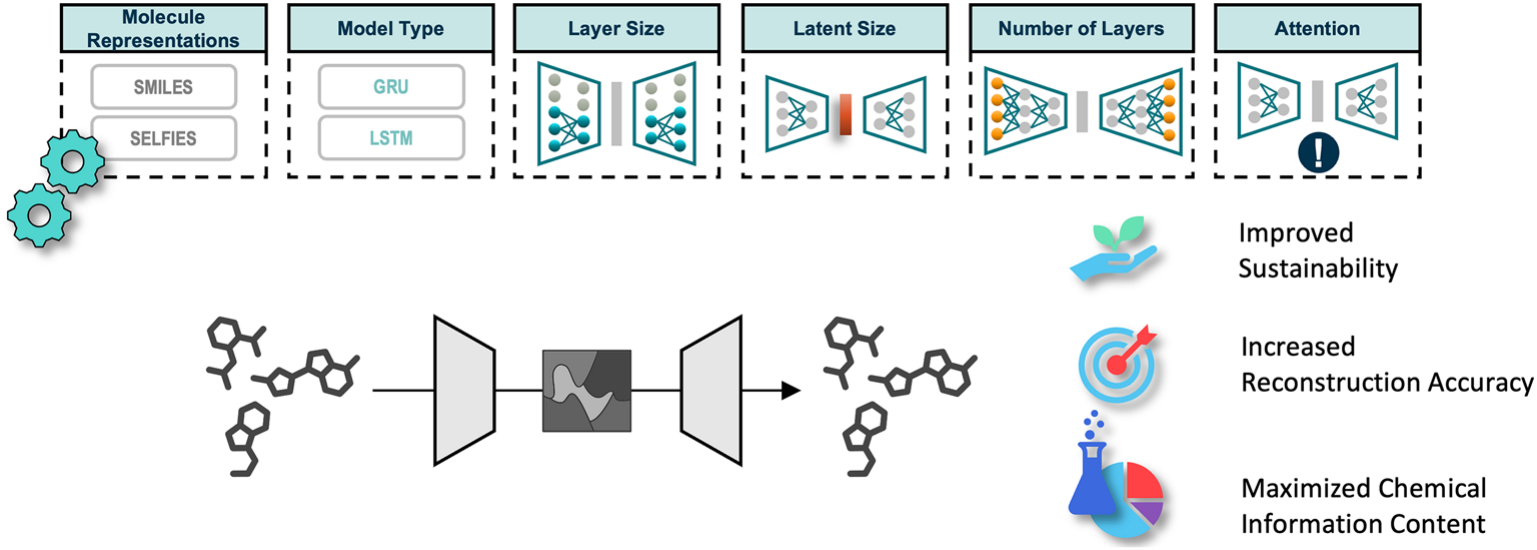

**Supplementary Information:**

The online version contains supplementary material available at 10.1186/s13321-024-00817-0.

## Introduction

The application of deep learning strategies on molecular data has drawn an increasing amount of attention in the last years [2–13]. The driving force behind this interest is the vastness of the chemical space and the impossible endeavor of exploring it manually [6, 14, 15]. Identifying a set of candidate molecules suitable for a given task is a slow process which usually only investigates fragments of the chemical space. Additionally, the areas that are investigated are typically selected in a highly biased manner by researchers. While an expert’s experience and knowledge are highly valuable resources during evaluation and finetuning of candidate molecules, the proposal of such candidates benefits from a computer’s capacity to traverse the entire molecule space in reasonable time. Thus, to move past selective exploration and to unlock the full potential of the chemical space, machine support, particularly deep learning, is appealing.

In order to train models on molecular data, suitable molecule representations are needed. Many formats exist to digitally represent molecules. One category of representations are string-based representations such as the simplified molecular-input line-entry system (SMILES) [16] and self-referencing embedded strings (SELFIES) [17].

SMILES were introduced in 1988 and are ubiquitously found in molecular databases. They are generated by traversing the molecular graph in a depth-first manner and denoting encountered atoms, bonds and other, higher-order structures with characters. However, the starting point for the traversal is not fixed and therefore various different SMILES can represent the same molecule. Like languages, SMILES not only have a defined vocabulary, but also a distinct grammar that regulates the token sequences. Hence, much like the human languages, a random combination of tokens is unlikely to result in a valid molecule. While the grammatical rules improve readability of this molecule representation, the invalidity of many token combinations has been stated as an issue in the context of generative machine learning applications [18]. Learning the underlying chemistry rather than focussing on the grammar of SMILES may be further facilitated by enumeration. Enumeration utilizes the mentioned feature that there exists not one but many SMILES for the same molecule. [12, 19].

Another string-based representation was introduced in 2020: SELFIES [17]. SELFIES guarantee validity by removing any kind of paired tokens as they exist in SMILES. Furthermore, the SELFIES format assures that every molecule can be represented and that any random combination of SELFIES tokens is valid. Like SMILES, also SELFIES are not not unique per molecule and can be enumerated.

However, these string-based representations are discrete and non-numeric, which stands in contrast to the continuous, numeric input preferred for use with deep-learning models.

As an alternative solution, autoencoders have been successfully used to first embed molecules and subsequently use the embeddings as molecular representations for downstream deep learning tasks [7–13, 20]. However, the architectures used are numerous and arbitrary. This makes the comparison of embeddings and quality control of the embedding space in general difficult, if not impossible.

Here, we systematically explore the impact of architectural changes on the performance of molecule autoencoders. Understanding the connection between architecture and performance is not only important for maximizing embedding quality, but also for optimizing the training procedure with respect to resource consumption. Training AI models requires increasingly specialized hardware and long training times. With growing model sizes it becomes incredibly expensive and thus almost exclusively accessible to large companies and wealthy countries. Additionally, the carbon dioxide footprint of AI models is alarmingly high: Strubell et al. [21] estimate that the emissions when training a large transformer network with neural architecture search amount to approximately 600 k pounds, which is 5-times as much as the emissions of an average car including fuel over its entire lifespan. In light of this, researchers are demanding solutions for greener and more publicly available AI models [21, 22]. We therefore assess how different architectural choices can affect the utility of the latent space generated by small molecule autoencoders, how this compares to the latent space of variational autoencoders (VAEs), and by what means these insights can be utilized to optimize the model architecture for high-quality small molecule embeddings while simultaneously reducing the resources spent on training. All models are made publicly available including open-source scripts to test and evaluate them as well as the option to train and evaluate custom architectures.

## Results

**Fig. 1:**
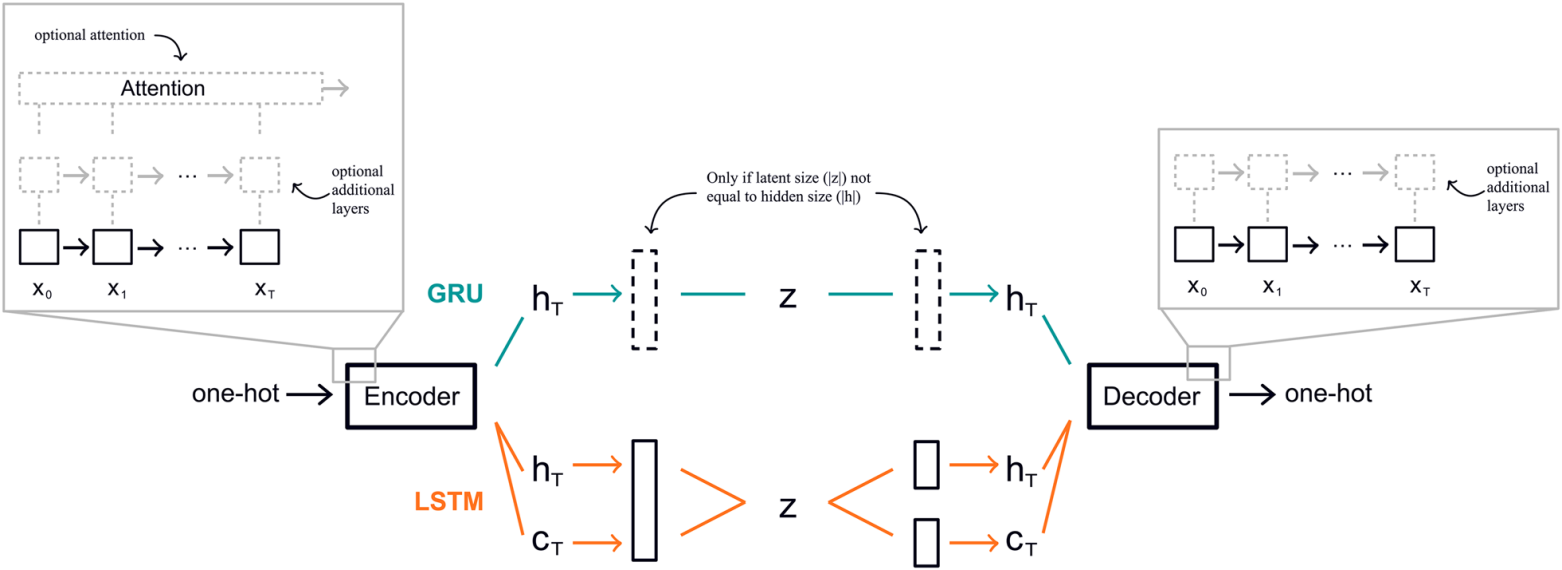
Architecture Overview. Illustration of the general autoencoder architecture used throughout the experiments. Molecules are provided and reconstructed as one-hot encodings of SMILES or SELFIES. The encoder and decoder are either GRUs or LSTMs. In case of GRUs (blue), the encoder returns the hidden state ${h}_{T}$ (after processing the last token of the input molecule), the size of which is controlled by the *hidden size*. If *latent size* and *hidden size* are not the same, an additional linear layer (dashed black rectangle) is introduced after the encoder GRU and before the decoder GRU. If *latent size* and *hidden size* are identical, then $z ={h}_{T}$. If the model is LSTM-based (orange), the encoder not only returns the last hidden state ${h}_{T}$ but also the last cell state ${c}_{T}$. They are concatenated and adapted to the latent size with an additional linear layer and to reconstruct hidden and cell state from the latents, two linear layers are introduced before the decoder (black rectangles). Both encoder and decoder may have additional layers and the encoder may further have an attention layer added to it (as illustrated in insets)

We have defined a base model architecture to serve as a reference throughout our experiments. This base model was implemented once as a Gated Recurrent Unit (GRU) and once as a Long Short-Term Memory (LSTM, for details see methods section) and they each comprised one layer, had a hidden size of 64, a latent size of 64 and did not use attention. The performances of the different architectures were then compared to these base models. Architectural parameters such as latent size, hidden size, number of layers and the use of attention were then systematically changed in separate experiments to evaluate their impact on the model’s performance in comparison to the base models. A general architectural overview including changeable parameters is given in Fig. 1. All models were trained on the MOSES [23] benchmarking dataset, both on the full set with 1.5 million training and 170,000 test molecules as well as on a small, random subset with 50,000 training and 12,500 test molecules which we will refer to as the 50 k subset. Choosing a subset was motivated by investigating the model’s performance when being provided substantially less training data and thus requiring less resources in terms of hardware and training time.

### Full reconstruction rate

The models were first assessed under data abundant conditions, i.e. trained on the full MOSES set and subsequently the performance was compared to the models trained on the much smaller subset. Specifically, we first assessed the Full Reconstruction rate, which is the percentage of test molecules that were correctly reconstructed in their entirety. Details on this metric can be found in the methods section.

#### Full set

**Fig. 2:**
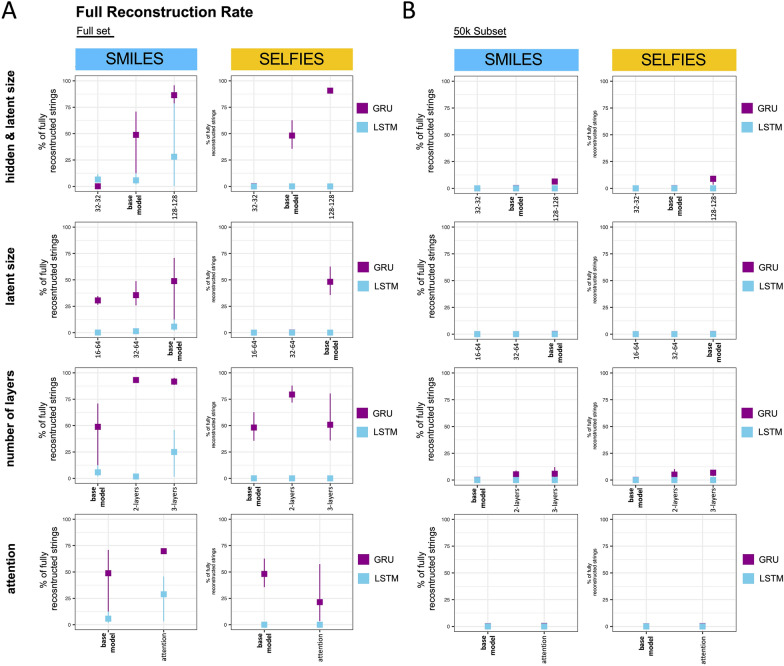
The effect of systematic adjustments of single architectural parameters on the Full Reconstruction rate. Shown is the Full Reconstruction on the test split achieved by models trained on the full set **A** and the 50 k subset **B** when adjusting hidden & latent size, only latent size, the number of layers and when adding attention. Each model was trained using three different seeds. The metric is presented as the mean of these seeds with error bars indicating the highest and lowest value. The performance of the base model (hidden and latent size of 64, one layer, no attention) is always shown as a reference to the modified architectures. GRU = Gated Recurrent Unit, LSTM = Long Short-Term Memory

As shown in Fig. 2A, across all models trained on the full set, GRUs generally outperform LSTMs in terms of Full Reconstruction.

When jointly adjusting *hidden and latent* size, both in SMILES and SELFIES, the GRU’s performance increased steadily, reaching around 90% in the biggest model assessed, while the LSTM performance stayed low, with only a slight improvement when pairing SMILES input with the biggest model of hidden and latent size 128.

The *latent size* limits the amount of information that can be carried in the latent space. Reducing it has memory-favorable effects but too small sizes might not hold enough information for reconstruction. To investigate whether the base model’s latent size of 64 can be further reduced without performance losses, it was decreased to 32 and 16. For GRUs with SMILES input, reduction to 32 reduced performance by 13%, but a further reduction to 16 only reduced performance slightly. In GRUs with SELFIES input on the other hand, reduction to 32 and 16 both lead to a total loss in Full Reconstruction. LSTMs had a poor starting performance on both input types so reducing the latent size further had barely any impact.

The next type of architectural modification that we investigated was the number of layers. When training GRUs on SMILES, performance drastically increased when adding a second layer, coming close to 100%. Therefore, adding a third layer had no further impact. When training GRUs on SELFIES, a second layer was also beneficial, but adding a third one reduced performance again to that of the base model. LSTMs trained on SMILES only benefitted from a third layer, while LSTMs trained on SELFIES did not show any improvement when adding a second or a third layer.

Lastly, when trained on SMILES, both GRUs and LSTMs increased performance when adding *attention*. When training on SELFIES, attention was not beneficial neither in GRUs nor LSTMs.

#### 50 k subset

As one of our leading questions was how the models compare in reconstruction performance when trained on a much smaller set, we assessed the Full Reconstruction rate of the same models trained on the 50 k subset. As illustrated in Fig. 2B, the Full Reconstruction rate dropped to zero or near zero across all scenarios.

### Mean similarity and Levenshtein distance

Measuring a model by only its fraction of perfectly reconstructed molecules is a harsh assessment, given that a single incorrect atom or bond would immediately lead to a Full Reconstruction rate of zero. Hence, a more detailed performance analysis is required in addition. Choosing the right metric for this depends strongly on the aspect to be assessed: From a molecular perspective, looking at the average number of correctly reconstructed tokens (Mean Similarity) between input molecule and the reconstructed molecule can provide meaningful insight. From a model perspective, a metric such as the Levenshtein Distance is of more interest. The reason is that RNNs generate their outputs sequentially based on previously observed tokens. For example, the insertion of an additional token that was not present in the original sequence would render all following tokens incorrect when measuring the Mean Similarity. Even if the sequence that follows the insertion is entirely correct and only shifted by one. The Levenshtein Distance, however, recognizes the correct reconstruction of the rest of the sequence and only penalizes the one incorrectly inserted token. For more details of these metrics, please refer to the methods.

**Fig. 3:**
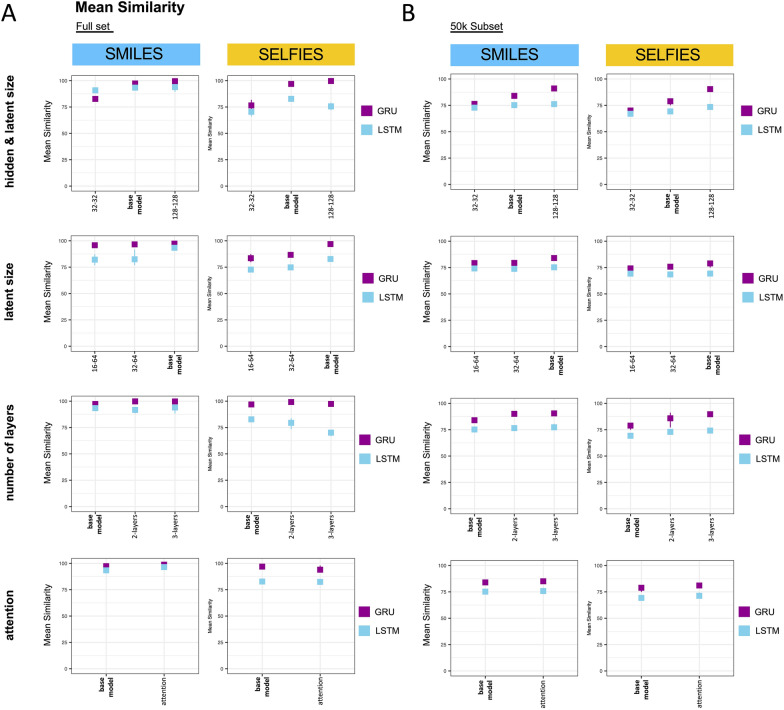
The effect of systematic adjustments of single architectural parameters on the Mean Similarity. Shown is the Mean Similarity reached on the test split achieved by models trained on the full set **A** and the 50 k subset **B** when adjusting hidden & latent size, only latent size, the number of layers and when adding attention. Each model was trained using three different seeds. The metric is presented as the mean of these seeds with error bars indicating the highest and lowest value. The performance of the base model (hidden and latent size of 64, one layer, no attention) is always shown as a reference to the modified architectures. GRU = Gated Recurrent Unit, LSTM = Long Short-Term Memory

In a first step, we assessed how much the Mean Similarity and Levenshtein Distance differed in the trained models. Here, for each trained model we computed both metrics between all test molecules and their reconstructions and averaged the metrics across all test molecules. We then compared the metrics across all models. Noticeably, Mean Similarity and Levenshtein Distance largely concur. Hence, the insights described for the Mean Similarity in the following are also mirrored in the Levenshtein Distance assessments (Additional file 1: Figure S1). Analogously to the previous section, we first evaluated the models trained on the full dataset (Fig. 3A) and then compared to those trained on the 50 k subset (Fig. 3B).

#### Full set

When modifying both *hidden and latent size*, GRUs trained on the full set show near perfect Mean Similarity both when trained on SMILES and SELFIES for the base model and the larger model with hidden- and latent-size of 128. LSTMs show comparable results as GRUs when trained on SMILES, however, when trained on SELFIES their performance is lower and there was no benefit from the larger architecture with hidden- and latent-size of 128.

When reducing the *latent size* only, GRUs trained on SMILES showed high Mean Similarity close to 100% on the base model and the models with reduced latent sizes. When training the GRUs on SELFIES, reducing latent size had a clear detrimental effect. LSTM performance slightly decreases when reducing latent size on both input representations.

Given the already outstanding performance in Mean Similarity of the GRU base model, GRUs trained on both input representations only benefited slightly from adding additional *layers*, however that slight improvement was clearly sufficient to push the number of correct Full Reconstructions as elaborated on above. LSTMs trained on SMILES had generally high scores in Mean Similarity with little benefit from adding additional layers. When LSTMs were trained on SELFIES, Mean Similarity reduced when a second or third layer was added.

GRUs and LSTMs trained on SMILES showed minimal improvements in Mean Similarity when adding *attention*, no positive effect was observed when both architectures were trained on SELFIES.

#### 50 k subset

Unlike what was observed in the Full Reconstruction rate, the Mean Similarity of the models trained on the 50 k subset was much more comparable to that of the models trained on the full set. Generally, the metric was high across models and molecular representations, approximating performance of the models trained on full set.

When changing both *hidden and latent size*, both GRUs and LSTMs trained on SMILES and SELFIES showed small improvements for the larger architecture and slight deterioration with the smaller architecture. Reductions in *latent size* had detrimental effects for GRU and LSTM trained on both SMILES and SELFIES, however, effects were minimal. Increasing the number of *layers* or adding *attention* had a minimal positive effect for both architectures and on both molecular representations.

In summary, the observations of the different metrics indicated that GRUs generally outperform LSTMs and SMILES were easier for the models to reconstruct than SELFIES. Additionally, while the Full Reconstruction drops drastically when training only on the 50 k subset, the Mean Similarity remains high in those models. Lastly, there is a strong agreement between the Mean Similarity, which only considers substitutions of tokens, and the Levenshtein metric which additionally considers insertions and deletions.

### Latent space utilization

Small latent sizes make it more difficult to store all the information required for reconstruction of the molecule. Increasing the latent size, however, inflates the size of the model and the memory needed during training and deployment, while not necessarily increasing model performance due to information saturation in the latent space. To assess how much the latent space of the models with varying latent sizes is utilized, a set of 12,500 test molecules was encoded by each of the models. For the 50 k subset, these comprised the entire 12,500 test molecules, while for the full set, where more than 170,000 test molecules were available, a subset of 12,500 was randomly sampled to make the results more comparable.

Depending on how the latent space is utilized, different utilization patterns can be observed that are visualized by a heatmap (Additional file 2: Figure S2A). Three utilization types can be observed in the models tested here: (1) *posterior collapse* [9]: Latent representations that look highly similar for a range of different molecules with low variance across latent dimensions; (2) *high utilization* represented by high variance in all dimensions, or (3) *Selective utilization* [9]: A blend of highly utilized latent dimensions and dimensions that are rarely utilized at all, which indicates that there are more dimensions available than necessary to store the information for reconstruction.

The GRU base model shows *selective utilization* on the subset and the full set, for both SMILES and SELFIES (Additional file 2: Figure S2B). GRU models with lower latent dimensions, i.e. 32 and 16, exhibit the *high utilization* latent space structure. On the other hand, none of the LSTM models trained on the subset exhibit *high utilization*: The base models exhibit *selective utilization* like their GRU counterparts but reducing the latent size shifts it towards *posterior collapse*, although small variances are still visible despite the prominent striping pattern of the heatmap (Additional file 2: Figure S2B). When trained on the full set, LSTM base models do not exhibit *selective utilization* (as the LSTM base model on the subset and the GRU base model on both sets), instead it demonstrates *high utilization*. Decreasing the latent sizes of these models prompts characteristics of *posterior collapse* as well as *high utilization* memory types and rather strong differences can be observed between the different seeds. The fact that the baseline model does not exhibit a selective memory structure indicates that increasing the latent size for these models may offer further room for improvement. Indeed, when increasing the latent size further to 128, the *selective utilization* structure becomes apparent (Additional file 2: Figure S2C). The *posterior collapse* of the LSTMs trained on the subset is less prominent when training on the full set.

### Subset-training optimization through increased training-time

**Fig. 4:**
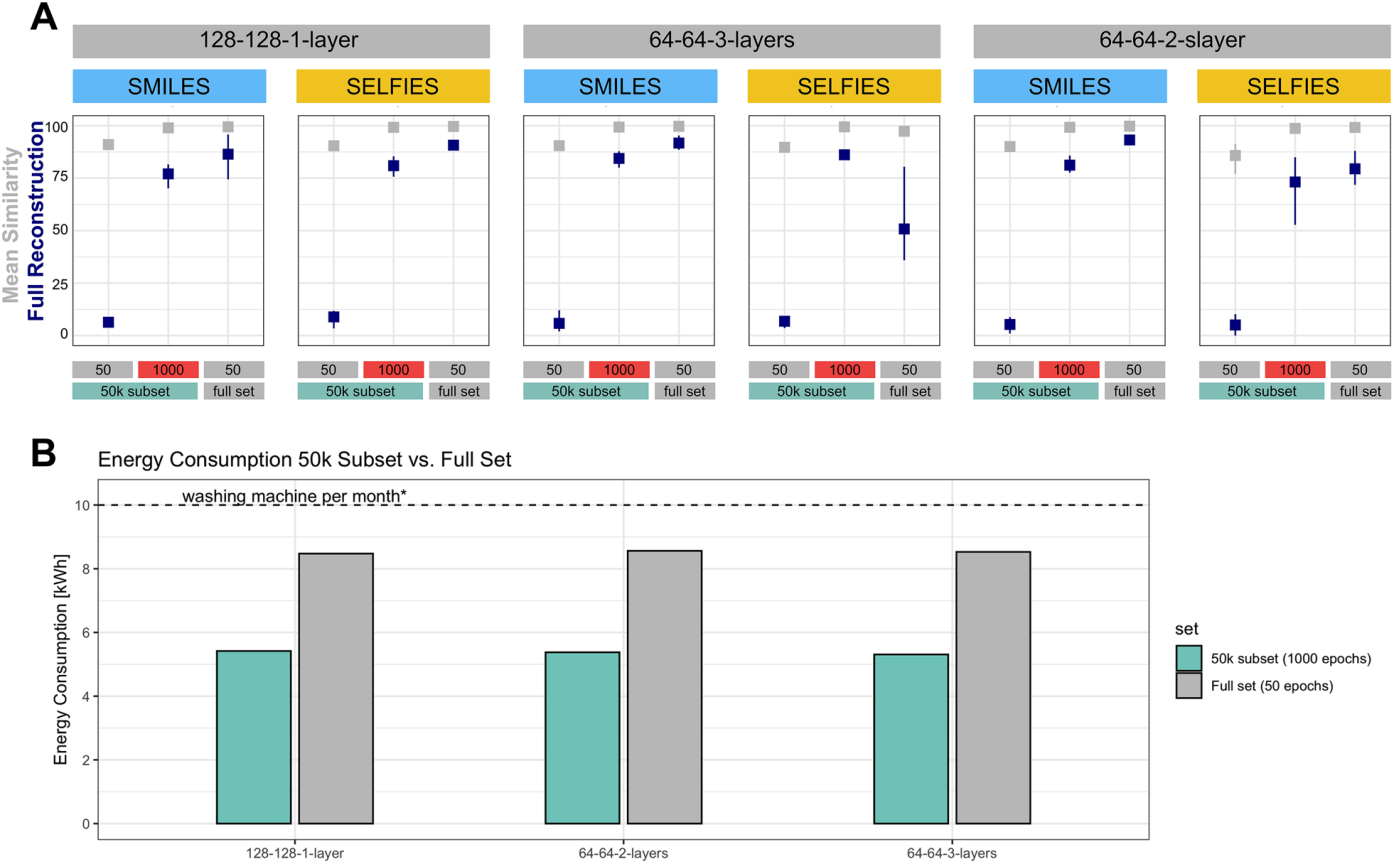
Subset performance optimization through train-time. **A**: Shown is the improvement of the models trained on the 50 k subset when increasing the training epochs from 50 to 1000 epochs and comparing to the performance of models trained on the full data set. The three architectures illustrated here were chosen because they were the best performing architectures in the experiments illustrated in Figs. 2, 3. Architectures are all GRUs without attention and model abbreviations follow the scheme “latent size—hidden size—number of layers”. Shown are Mean Similarity (gray) and Full Reconstruction (blue) on the test split. The metrics are presented as the mean of three seeds with error bars indicating the highest and lowest value. **B**: Energy consumption of the three models trained on the full set for 50 epochs and the 50 k subset for 1000 epochs, which achieved comparable reconstruction performance on the test set. *The average energy consumption of using a washing machine in Europe is estimated at 120 kWh per year [24], i.e., 10 kWh per month

The experiments above illustrated general inferiority of the models trained on the 50 k subset in comparison to the full set. However, this deficit was mostly rooted in the much poorer Full Reconstruction rate while the Mean Similarity was comparable. We therefore investigated if training the models on the 50 k subset for more epochs could rescue the Full Reconstruction metric in a similar way as training it on the full dataset. We selected the three best performing models in both metrics and both string representations: [1] GRU, latent size 128, hidden size 128, one layer, no attention; [2] GRU, latent size 64, hidden size 64, three layers, no attention; [3] GRU, latent size 64, hidden size 64, two layers, no attention. We then trained them on the 50 k subset for 1000 epochs in order to assess whether the performance issues can be mitigated by longer training. Longer training indeed improved the models drastically, boosting all their Full Reconstruction rates beyond 70%, while reaching near perfect Mean Similarity (Fig. 4A). Although most of the models trained on the full dataset remained slightly better than those trained on the 50 k subset for 1000 epochs, longer training clearly compensated for the much lower sample size (97% less data than in the full set). It is noteworthy that the training on the full set for 50 epochs consumed around 8.5 kWh for all three architectures, while training on the 50 k subset for 1000 epochs—and thus reaching a comparable final performance—required only around 5.4 kWh (Fig. 4B). In comparison, the energy consumption of a household’s washing machine per month in Europe is approximately 10 kWh [24]. In terms of produced CO_2_, in Germany 8.5 kWh equate to 3.7 kg of CO_2_ using emissions of energy production in 2022 [25].

### Subset-training optimization through additive optimization

Following the results from the previous experiments, the next step was to assess if the observations made in the separate experiments allow for an additive optimization of the architecture. We selected the best setting from each experiment for SMILES and SELFIES and built a model combining all these architectural choices. We then tested, if the performance of those inferred best models outperformed the three best models in the singular experiments (1) GRU, latent size 128, hidden size 128, one layer, no attention; (2) GRU, latent size 64, hidden size 64, three layers, no attention; (3) GRU, latent size 64, hidden size 64, two layers, no attention). For SMILES, GRUs outperformed LSTMs, a hidden and latent size of 128 performed best, three layers were better than one or two layers and attention improved the performance. Thus, the inferred best model for SMILES was: GRU, latent size 128, hidden size 128, three layers, with attention.

For SELFIES, GRUs also outperformed LSTMs and the same hidden and latent size as in the SMILES experiment achieved the best performance. However, three layers either brought only small improvements or even significantly decreased the performance for some of the cases. Like in the SMILES case, attention slightly improved the performance in the 50 k subset. Hence, the inferred best model chosen for SELFIES was: GRU, latent size 128, hidden size 128, two layers, with attention.

**Fig. 5:**
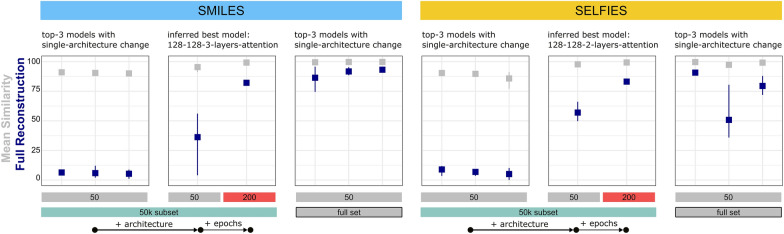
Effect of additive optimization. Shown on the left and right as a reference are the best performing architectures identified during the systematic adjustment of single architectural parameters, once trained on the 50 k subset (left) and the full set (right). The center shows the performance of the additively optimized models, for each SMILES and SELFIES trained on the 50 k subset for 50 epochs as well as 200 epochs (the latter only for the best performing seed from the 50 epoch experiments). Architectures are all GRUs and model abbreviations follow the scheme “latent size—hidden size—number of layers—use of attention”. Shown are Mean Similarity (gray) and Full Reconstruction (blue) on the test split

For both SMILES and SELFIES, the inferred best model trained on the subset for 50 epochs outperformed the top 3 models from the singular experiments, especially in terms of Full Reconstruction rate (Fig. 5). When continuing the training of the inferred best models for a total of 200 epochs for the best performing seed, both Full Reconstruction rate and Mean Similarity approached or surpassed those of the top 3 models from the singular experiments that were trained on the full set. This demonstrates that performance gain is possible by additive optimization.

### Latent space evaluation

While the models achieve high reconstruction performance in both Mean Similarity and Full Reconstruction after additive optimization, they are not variational autoencoders (VAEs) and therefore do not apply any explicit constraints onto the latent space. We thus analyzed how well chemical similarity is reflected in their latent spaces and how that compares to the latent space of a VAE. For the non-variational autoencoders, we additionally investigated the effect of enumeration of the input or the target molecule, motivated by the observations that SMILES enumeration enhances the latent space of autoencoders [12]. Here, enumeration effects were also investigated for SELFIES. We analyzed the latent space of eight models (Table 1).

**Table 1:** Model architectures considered for latent space evaluation

Name	Variational autoencoder	Architecture	Molecule representation	Enumerated
SMILES-AE-can2can	No	GRU, 128 hidden and latent size, 3 layers, attention	SMILES	No
SMILES-AE-enum2can	No	GRU, 128 hidden and latent size, 3 layers, attention	SMILES	Yes (input)
SMILES-AE-can2enum	No	GRU, 128 hidden and latent size, 3 layers, attention	SMILES	Yes (output)
SMILES-VAE-can2can	Yes	GRU, 128 hidden and latent size, 3 layers, attention	SMILES	No
SELFIES-can2can	No	GRU, 128 hidden and latent size, 2 layers, attention	SELFIES	No
SELFIES-enum2can	No	GRU, 128 hidden and latent size, 2 layers, attention	SELFIES	Yes (input)
SELFIES-can2enum	No	GRU, 128 hidden and latent size, 2 layers, attention	SELFIES	Yes (output)
SELFIES-VAE	Yes	GRU, 128 hidden and latent size, 2 layers, attention	SELFIES	No

Architectures are chosen based on the results of the additive optimisation for SMILES and SELFIES

**Fig. 6:**
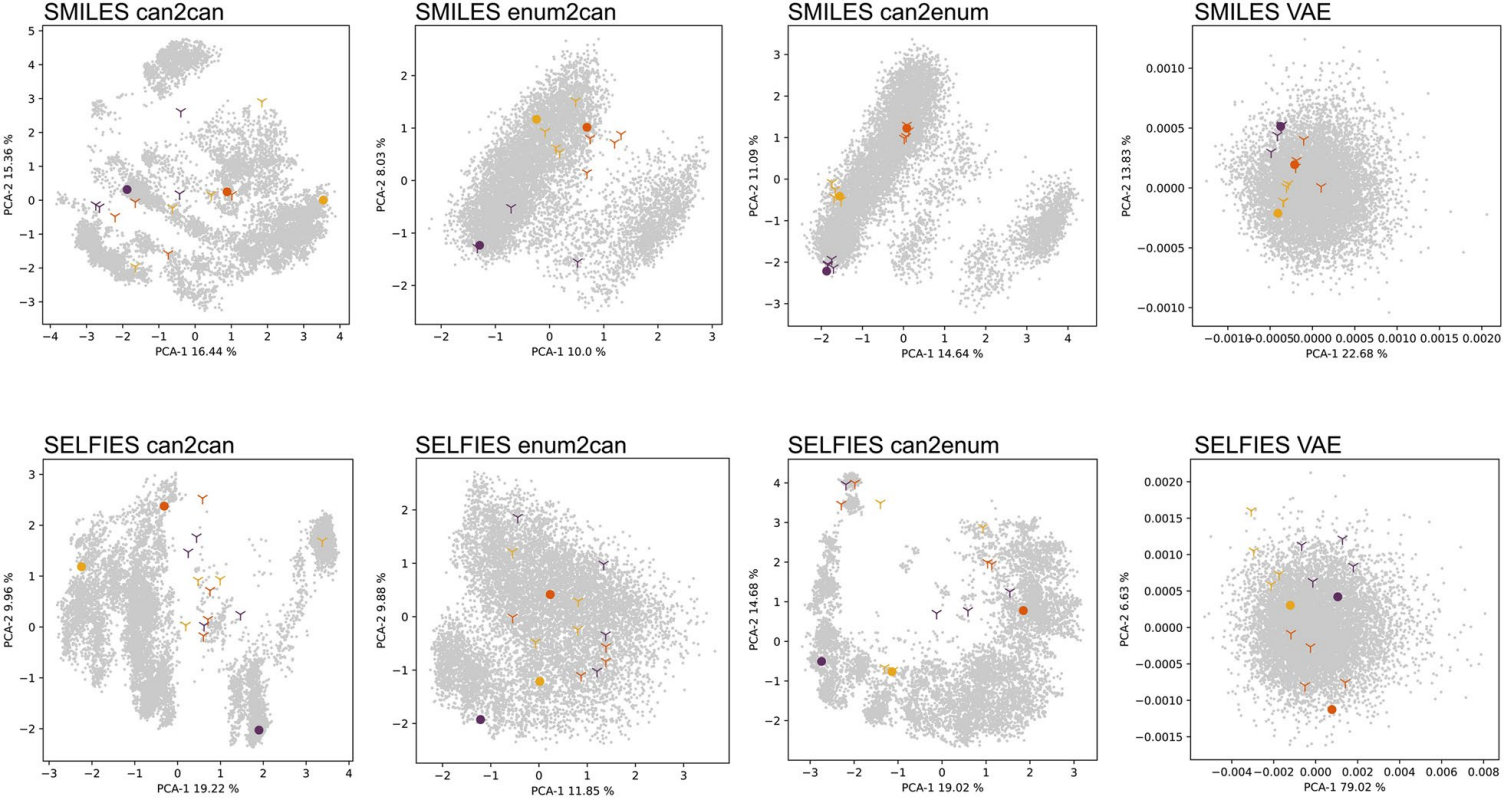
Latent space evaluation for chemical similarity. The PCAs show the latent representations of 10,000 random molecules (gray) and three test molecules (coloured dots). Enumerated versions of the test molecules are represented as crosses. Every panel represents a different model as indicated above. The percentages of each principal component indicate the amount of variance that they represent

All models were trained for 200 epochs and on the 50 k subset. For details on the evaluation, please refer to the methods section. As illustrated in Fig. 6, the *SMILES-AE-can2can* model’s latent space poorly reflected molecular similarity. Enumerated versions of the same SMILES were mapped to vastly different areas of the latent space and did not cluster with the canonical SMILES of the same molecule. The latent space showed low coherence, an issue associated with non-variational autoencoders which can often be mitigated by using VAEs. The homogeneity of the latent space increased when training the model with enumerated SMILES as input and canonical SMILES as targets, nonetheless it is separated into two high-density areas and not fully homogeneous. While the embedding overall remains poor, the embeddings of the enumerations co-located more than in the can2can model. Training on canonical SMILES as input and enumerating the target SMILES improved this, with the enumerations now clustering closely to the canonical representations of the same molecules. Implementing the model as a VAE (*SMILES-VAE-can2can*) instead of an AE (*SMILES-AE-can2can*) expectedly lead to a more consistent latent space, nonetheless, some of the enumerations scatter away from their canonical counterparts.

Much like in the SMILES case, the latent space of the AE trained on canonical SELFIES only was incoherent and enumerations were located far apart from their canonical counterparts. Unlike in the SMILES case, training the SELFIES-based models on enumerations did not benefit the co-localization of enumerated SELFIES. However, the latent space did become more coherent, especially in the enum2can model.

In the SELFIES VAE, the latent space expectedly becomes more regular and while the enumerations of the three test molecules are not overlapping each other, co-localization of canonical SELFIES and their enumerations is not optimal. However, while the latent space of the VAEs is shaped more favorably, their stochastic nature leads to low reconstruction performance: a Mean Similarity of 77.3% for SMILES and 79.3% for SELFIES, as well as a 0% Full Reconstruction for SMILES and 0% for SELFIES, with the Kullback–Leibler divergence-term weighted with 0.3 and therefore already favoring reconstruction accuracy.

These observations are further supported when looking at Euclidean distances between latent representations of molecules within a group, i.e. the reference molecule and its four enumerations, compared to that of random molecules (Additional file 3: Figure S3). The distinction is particularly prominent for the *SMILES-AE-can2enum* model.

## Discussion

In this work, we systematically assessed how model architecture as well as molecular input format impact not only the reconstruction performance of a molecular autoencoder, but also the organization and quality of its latent space. This was motivated by the need for a better understanding of how we can carefully engineer and optimize the architecture to reduce the resources required during training while simultaneously maximizing encoding quality. The experiments have shown that when comparing RNNs, GRUs generally outperform LSTMs when encoding and reconstructing string representations of molecules. While in another work, Chung et al. [26] found no difference in performance between GRUs and LSTMs when encoding and decoding audio sequences, their models were designed such that despite the different number of gates, they had approximately the same number of parameters, which was neither done, nor desired in this work. Models with more parameters are known to be more prone to overfitting when trained on the same set as a model with less parameters. This could explain the LSTM’s inferiority in our case. Additionally, Chung et al. [26] trained on much longer sequences than the SMILES and SELFIES used here and therefore illustrate the behavior of GRUs and LSTMs for different length categories. As a future direction, graph neural networks (GNNs) should be included in this comparison, utilizing molecular graphs as input rather than string formulations of molecules. However, while more and more architectures for GNNs are being explored, high-quality end-to-end solutions that not only allow encoding but also decoding of graphs including all the essential chemical properties such as atom- and bond-types, chemical rules and charges are yet missing.

We also observed that SELFIES were harder to correctly reconstruct than SMILES for most of the models tested here. While this may be surprising at first given their proposed advantages for deep learning, upon further consideration it is plausible: SELFIES are per definition always valid, even when constructed at random, which does not provide the model any syntactic rules to learn. In SMILES, on the other hand, there are some token combinations that the model would never encounter because they simply do not represent valid chemistry. Additionally, unlike in SMILES, the tokens that make up SELFIES do not always have the same meaning. Instead, they are rules that represent which bonds, atoms, rings or branches are valid next pieces given what the molecule looks like so far. The mentioned absence of invalid examples paired with this equivocality of the tokens may explain why it is harder for the models to reconstruct SELFIES than it is to reconstruct SMILES. The lack of coherent grammar may also explain why enumeration does not have the rescuing effect observed in SMILES in terms of latent space organization. Other works have pointed out that the encoding system of SELFIES with its roots in theoretical computer science in some application scenarios makes them more easily understandable for computers than other string formats [27, 28]. However, in the particular scenario investigated in this study, asides from their guaranteed validity, SELFIES do not outperform SMILES. Nonetheless, the evaluation of the models as performed here has a strong statistical focus. Future investigations with a focus on chemistry may help evaluate the models further. Particularly, how well suited are the latent spaces to train for instance QSAR models and how present are undesired chemical structures in molecules sampled from the latent space.

The combined results in the models trained on the 50 k subset, where the Full Reconstruction was near zero but the Mean Similarity was distinctively high and comparable to that of the models trained on the full set, demonstrate that the extremely low Full Reconstruction in the subset is due to few incorrectly reconstructed tokens rather than complete reconstruction failure. The experiments also showed that careful architecture design strongly impacts both reconstruction performance and resource consumption, specifically, that when optimizing the architecture, good reconstruction performance could be achieved using only 3% of the data and reducing energy consumption by around 36% compared to a non-optimized architecture trained on the full set. This firstly implies that the concerningly high resource consumption of training machine learning models can be counteracted by careful architecture design offering a step towards realizing green AI. And secondly, achieving comparable results on much less data when carefully designing the model’s architecture allows training on less specialized hardware. This is an important step towards making AI broadly available and therefore enabling the democratization of AI.

## Conclusion

The results of this work demonstrate that high model quality and low resource consumption are not mutually exclusive, but that they can be harmonized by careful architecture design. It is clearly illustrated that the chemical information content of autoencoder latent spaces can be maximized to provide more chemically meaningful input to downstream applications, while simultaneously moving towards greener AI models that use significantly fewer data points and consume less energy during training. This in turn makes the development of these models less dependent on highly specialized hardware and contributes to the democratization of AI. However, more research is required with regards to how this can be done efficiently. The computational overhead of training the variety of models presented here to engineer an optimized architecture does not go hand in hand with the idea of saving resources. A better understanding of the systematic connections between architecture and the machine learning task must be acquired and additionally translated to other model types to make this optimization process efficient and sustainable in the future. This falls into the field of explainable AI and is a crucial step towards saving energy and democratizing AI by building a suitable model for a specific task rather than the frequently encountered approach of compensating generic model design with larger data and longer training.

## Methods

### Datasets and molecule representations

To train our models, we used the benchmarking dataset molecular sets (MOSES) [23]. This benchmarking set comes with a provided train-test-split of approximately 1.5 million molecules for training and approximately 170,000 molecules for testing. The dataset is based on the ZINC Clean Leads which were then filtered further for different criteria such as contained atom types, maximum allowed ring sizes and charges, as described in the original paper [23].

Given the large amount of energy that is consumed when training machine learning models and the devastating impact it has on the environment, energy-conscious or—in the best case—green computing is an important topic. To this end we created an additional, much smaller random subset of the MOSES dataset, comprising only 50,000 training molecules and 12,500 test molecules. We then used this subset to compare the achieved model qualities to those trained with the full dataset in order to explore the trade-off between smaller training time with the subset and performance boost with the full set. The token frequencies and string length distribution of the subset are representative of that of the full set (Additional file 4: Figure S4).

In order to explore the impact that different molecule representations have on the embedding quality of the autoencoders, we selected two different string representations: SMILES and SELFIES. The SMILES representations were obtained directly from the MOSES dataset. For the enumeration experiments, we used a 5x-enumeration (one canonical, four non-canonical SMILES) based on the enumeration method provided by https://github.com/EBjerrum/SMILES-enumeration. SELFIES were constructed from SMILES (and their enumerations) using the *selfies* python library (version 2.1.1).

### Models

The models used here are recurrent neural network (RNN)-based models with string input. More specifically, we compared gated recurrent unit (GRU) and long short-term memory (LSTM) architectures. For each, GRU and LSTM, we chose a base architecture to which we iteratively applied changes and investigated their impact. The base architectures were 1) GRU, one layer, hidden size 64, latent size 64, no attention and 2) LSTM, one layer, hidden size 64, latent size 64, no attention. 64 was chosen as the latent and hidden size in the base model because it exceeds the maximum sequence length in the dataset and therefore does not require any dimensionality reduction by the model. We then investigated the impact of jointly changing the hidden and latent size. For that we explored a reduction in size (32 hidden size, 32 latent size) as well as an increase (128 hidden size, 128 latent size). To further unravel the impact of each, we then added experiments where the hidden size was fixed to that of the base model and only the latent size was altered to either 32 or 16. Additionally, we inspected the impact of adding additional layers, comparing RNNs of each category with one, two and three layers. Lastly, the impact of attention on the model performance was examined in both the GRU as well as the LSTM case. See Additional file 5: Table S1 for a comprehensive list of experiments.

If not explicitly stated otherwise, the models were trained for at least 50 epochs. Early stopping was set up to act after the minimum of 50 epochs were reached and limited to a maximum of 500 epochs. The early stopping was configured to a minimum delta in validation loss of 0.01 with a patience of 5. An adam optimizer with learning rate of 0.005 was used. Teacher forcing was applied to avoid accumulative errors when predicting incorrect tokens. We used a cross-entropy loss and all models were trained for three different seeds and the seeds were the same across models. The reported results are mean values from these three runs.

For GRUs, the latent representation is the model’s hidden state after processing the last input token. However, since LSTMs return not only the hidden but also the cell state, they were first concatenated and then passed to a linear layer to create the latent representation in order to avoid a doubling in size. The decoder’s hidden state was initialized with the latent representation in case of GRUs. For LSTMs, two linear layers were implemented to reconstruct the hidden and the cell state from the latent representation, which then initialized the LSTM’s decoder.

### Evaluation metrics

#### Mean Similarity

For all input and subsequently reconstructed molecules, the average number of correctly reconstructed tokens is calculated. Concisely, the Mean Similarity of a molecule to its reconstruction is.

$${\text{Mean Similarity}} = \,\frac{1}{{\text{n}}}\mathop \sum \limits_{{{\text{i}} = 1}}^{{\text{n}}} {\text{f}}\left( {{\text{s}}_{{\text{i}}}^{{{\text{input}}}} ,{\text{~~s}}_{{\text{i}}}^{{{\text{reconstructed}}}} } \right)$$,

with $n$ the string length in tokens and ${{\text{s}}}^{{\text{input}}}$, ${{\text{s}}}^{{\text{reconstructed}}}$ the input and reconstructed string, respectively. $f({s}_{i}^{input},{s}_{i}^{reconstructed})=1$ if ${s}_{i}^{input}={s}_{i}^{reconstructed}$ and 0 otherwise. The Mean Similarity across all test molecules is then averaged for each model.

This is the easier metric, since rare and difficult to reconstruct tokens do not impact this metric severely.

#### Levenshtein distance

The Levenshtein Distance, also referred to as the edit distance, states the minimum number of tokens that need to be changed to transform one string into another [29]. This includes substitutions, insertions and deletions. To make this comparable to the other two metrics considered here, which are similarities, we computed $1-\frac{LD}{n}$, with $n$ the string length in tokens.

#### Full reconstruction

For all input and subsequently reconstructed molecules, the Full Reconstruction rate of a model is the percentage of test molecules that were perfectly reconstructed, i.e., without a single mismatch.

This metric is more difficult since it also captures the model’s ability to learn rare tokens.

### Latent-Space analysis

If downstream machine learning models are to be trained on the autoencoder’s latent space, a good reconstruction accuracy is not the only important factor: The latent space itself must also reflect chemical similarity with similar molecules being located in close proximity and different molecules further apart. To evaluate the latent space, three randomly chosen SMILES from the test set were enumerated four times, i.e. four other SMILES representing the same molecule were created, yielding 5 different SMILES for each of the three test molecules. For the evaluation of SELFIES-based models, these SMILES were then translated to yield enumerated SELFIES. They were then embedded with the model’s encoder. Two tests were performed: (1) A principal component analysis (PCA) was performed on the embeddings of the enumerated SMILES as well as 10,000 random molecules and their spatial arrangement was visualized based on the first two principal components. (2) For each of the three test molecules, the average Euclidean distance of their embeddings was calculated. Then, 1000 random molecules were embedded and their pairwise Euclidean distances were calculated and plotted as a histogram together with the mean distances of the test molecules.

### Computing resources

All experiments were run using four NVIDIA Tesla V100 GPUs each with 32 GB memory. Energy benchmarking was performed on the HAICORE@KIT partition using four NVIDIA A100 GPUs with 40 GB memory each.

### Supplementary Information

**Additional file 1: Figure S1.** The effect of systematic adjustments of single architectural parameters on the Levenshtein Distance. Shown is the average Levenshtein Similarity (1—average Levenshtein Distance) reached on the test split achieved by models trained on the full set **A** and the 50 k subset **B** when adjusting hidden & latent size, only latent size, the number of layers and when adding attention. Each model was trained using three different seeds. The metric is presented as the mean of these seeds with error bars indicating the highest and lowest value. The performance of the base model (hidden and latent size of 64, one layer, no attention) is always shown as a reference to the modified architectures. *GRU* gated recurrent unit, *LSTM* long short-term memory.

**Additional file 2 Figure S2. **Latent space utilization. The molecule encodings were scaled to a range of [0, 1] using min-max scaling, to make patterns comparable across models when visualized as a heatmap. Rows represent molecules and columns represent latent dimensions. **A** Three types of latent space utilization patterns were found in the models. Left: *posterior collapse* type, characterized by low variance in the latent dimensions and encodings that show high resemblance. There is little information content for reconstruction. Middle: *high utilization* type, characterized by high variance in the latent dimensions, notable differences between the encodings and high information content. Right: *selective* type, characterized by high variance in most but very low variance in some latent dimensions and showing saturation of carried information content. **B** Latent space utilization is visualized as heatmaps when modifying the size of the latent vector. The top two rows show results when models were trained on the subset, the bottom two rows show results for the full MOSES set. The three leftmost columns represent models trained on SMILES, the three rightmost columns are models trained on SELFIES. Rows that represent GRU and LSTM models are marked as such on the left. Latent space sizes are stated on top. For each model, three latent space utilization plots are shown, representing the three different seeds used for training. **C** Latent space utilization of LSTMs trained on the full MOSES dataset with latent size 128. Three latent space utilization plots are shown for each SMILES and SELFIES, representing the three different seeds used for training

**Additional file 3 Figure S3.** Euclidean distances of latent representations from similar versus random molecules. For each investigated model, the Euclidean distances of the latents of 1000 random molecules is illustrated as a histogram (blue). For the three test molecules, the average Euclidean distance between the original and its four enumerations are indicated by coloured vertical lines.

**Additional file 4 Figure S4.** String length distributions and token frequencies in the full set and the subset. **A** shows the string length distribution (left) and the token frequencies (right) of SMILES for both the full MOSES set (turquoise) and the subset (orange). **B** illustrates this for SELFIES. The token frequencies are normalized to the number of molecules in the set.

**Additional file 5 Table S1.** Overview of the experiments used for the systematic assessment of single architectural parameters. In bold are architecture features that were modified in comparison to the base models. Arrows indicate if the feature was increased or decreased in comparison to the base model.

## Data Availability

All data used in this work comes from publicly available databases. All trained models including the development code and data are provided at Zenodo: https://zenodo.org/records/10664803.

## Abbreviations

PCA: Principal component analysis
GRU: Gated recurrent unit
LSTM: Long short-term memory
RNN: Recurrent neural network
MOSES: Molecular sets
GNN: Graph neural network
SMILES: Simplified molecular-input line-entry system
SELFIES: Self-referencing embedded strings
VAE: Variational autoencoder
AE: Autoencoder

## Code availability

The source code and top models can be found on GitHub with scripts to encode custom molecules: https://github.com/MarieOestreich/small-molecule-autoencoders.
