# Supplementary figures and images for: Small molecule autoencoders: architecture engineering to optimize latent space utility and sustainability
Source: J Cheminform. 2024 Mar 5;16:26. doi: 10.1186/s13321-024-00817-0 (PMC10913550; doi:10.1186/s13321-024-00817-0)

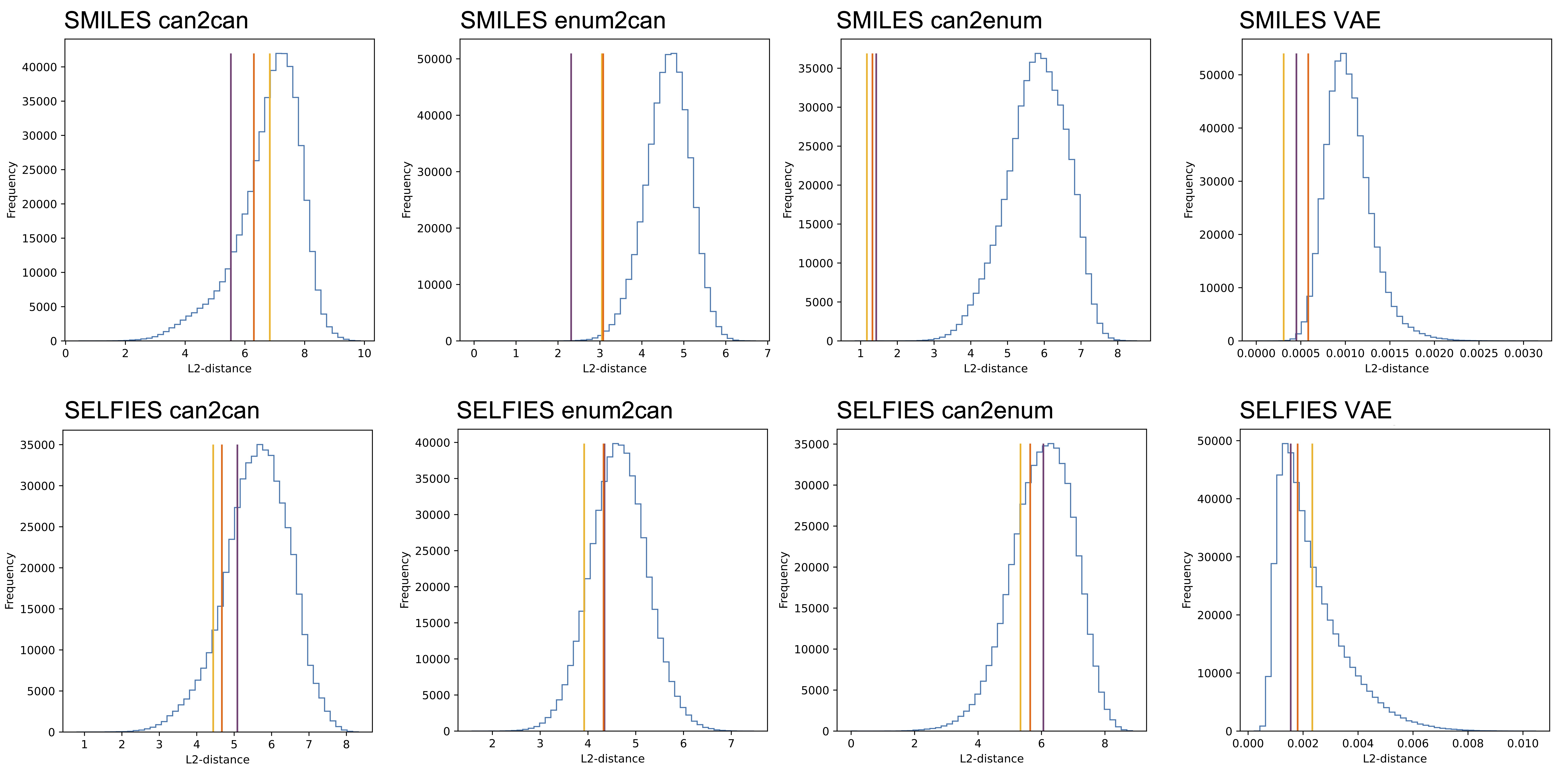

Supplement: Supplementary file 3 — Additional file 3 Figure S3. Euclidean distances of latent representations from similar versus random molecules. For each investigated model, the Euclidean distances of the latents of 1000 random molecules is illustrated as a histogram (blue). For the three test molecules, the average Euclidean distance between the original and its four enumerations are indicated by coloured vertical lines. [file 13321_2024_817_MOESM3_ESM.jpg]

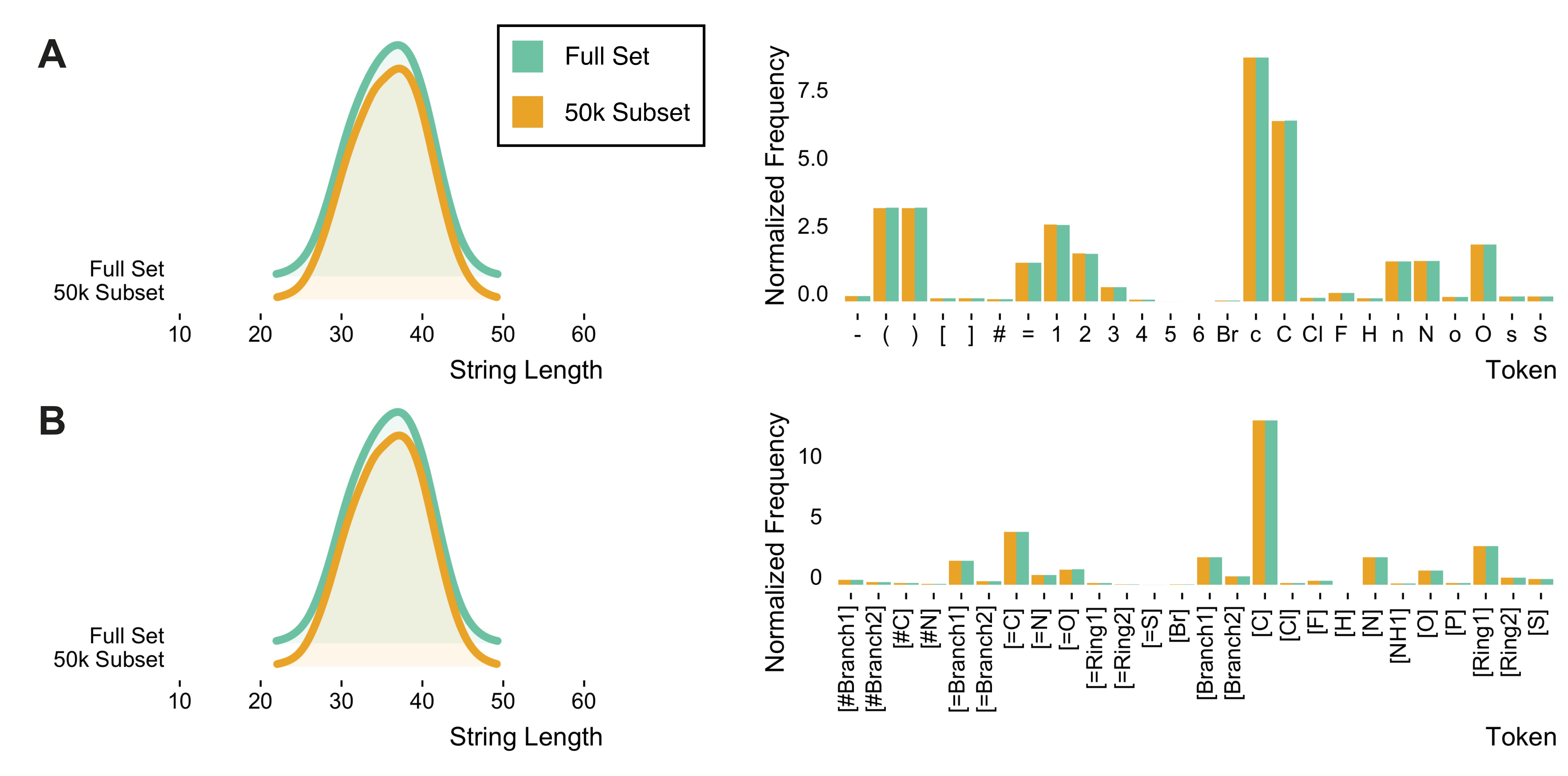

Supplement: Supplementary file 4 — Additional file 4 Figure S4. String length distributions and token frequencies in the full set and the subset. A shows the string length distribution (left) and the token frequencies (right) of SMILES for both the full MOSES set (turquoise) and the subset (orange). B illustrates this for SELFIES. The token frequencies are normalized to the number of molecules in the set. [file 13321_2024_817_MOESM4_ESM.jpg]

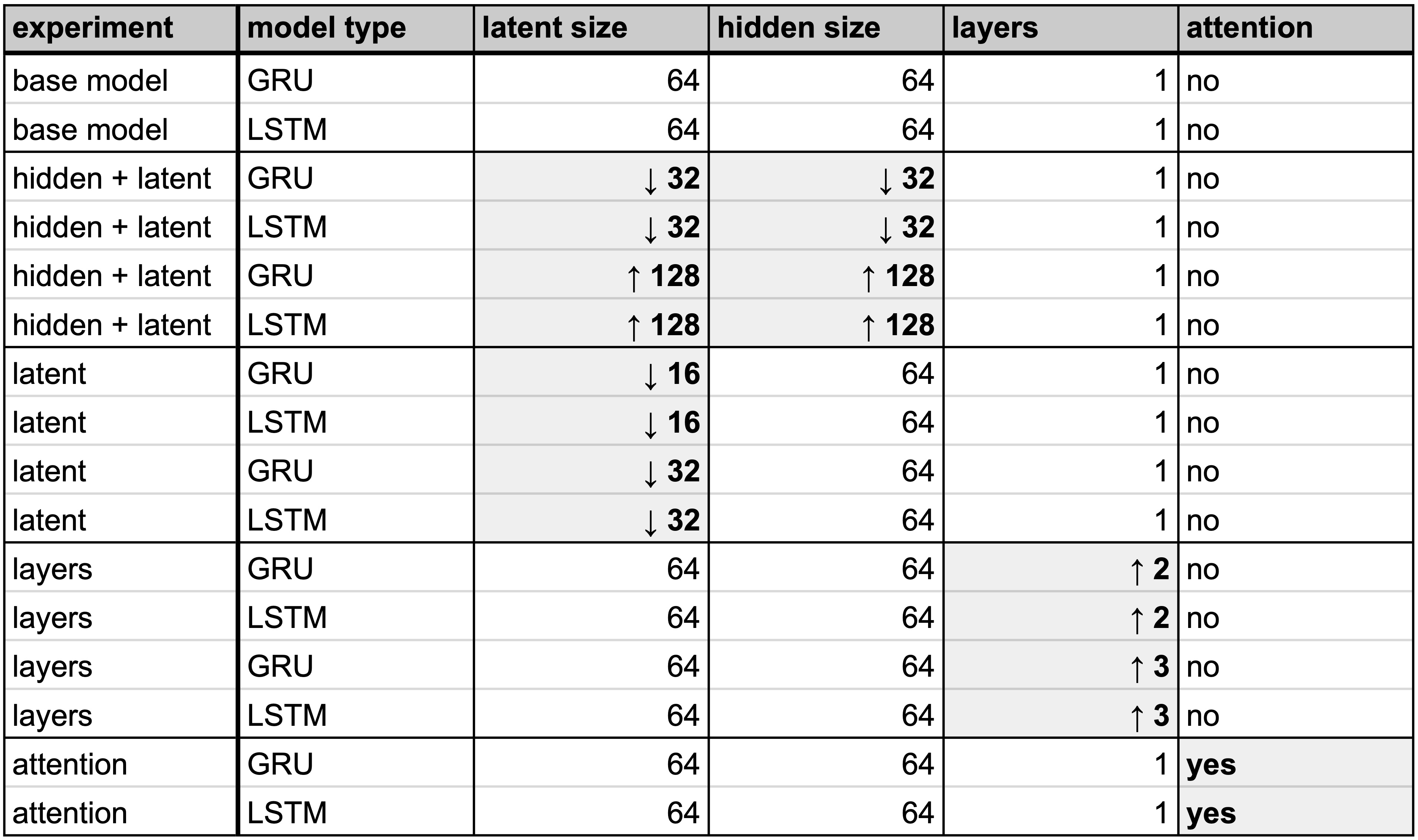

Supplement: Supplementary file 5 — Additional file 5 Table S1. Overview of the experiments used for the systematic assessment of single architectural parameters. In bold are architecture features that were modified in comparison to the base models. Arrows indicate if the feature was increased or decreased in comparison to the base model. [file 13321_2024_817_MOESM5_ESM.jpg]
